# Automated prostate cancer detection via comprehensive multi-parametric magnetic resonance imaging texture feature models

**DOI:** 10.1186/s12880-015-0069-9

**Published:** 2015-08-05

**Authors:** Farzad Khalvati, Alexander Wong, Masoom A. Haider

**Affiliations:** Department of Medical Imaging, University of Toronto, Toronto, ON Canada; Physical Sciences, Sunnybrook Research Institute, Toronto, ON Canada; Department of Systems Design Engineering, University of Waterloo, Waterloo, ON Canada

## Abstract

**Background:**

Prostate cancer is the most common form of cancer and the second leading cause of cancer death in North America. Auto-detection of prostate cancer can play a major role in early detection of prostate cancer, which has a significant impact on patient survival rates. While multi-parametric magnetic resonance imaging (MP-MRI) has shown promise in diagnosis of prostate cancer, the existing auto-detection algorithms do not take advantage of abundance of data available in MP-MRI to improve detection accuracy. The goal of this research was to design a radiomics-based auto-detection method for prostate cancer via utilizing MP-MRI data.

**Methods:**

In this work, we present new MP-MRI texture feature models for radiomics-driven detection of prostate cancer. In addition to commonly used non-invasive imaging sequences in conventional MP-MRI, namely T2-weighted MRI (T2w) and diffusion-weighted imaging (DWI), our proposed MP-MRI texture feature models incorporate computed high-b DWI (CHB-DWI) and a new diffusion imaging modality called correlated diffusion imaging (CDI). Moreover, the proposed texture feature models incorporate features from individual b-value images. A comprehensive set of texture features was calculated for both the conventional MP-MRI and new MP-MRI texture feature models. We performed feature selection analysis for each individual modality and then combined best features from each modality to construct the optimized texture feature models.

**Results:**

The performance of the proposed MP-MRI texture feature models was evaluated via leave-one-patient-out cross-validation using a support vector machine (SVM) classifier trained on 40,975 cancerous and healthy tissue samples obtained from real clinical MP-MRI datasets. The proposed MP-MRI texture feature models outperformed the conventional model (i.e., T2w+DWI) with regard to cancer detection accuracy.

**Conclusions:**

Comprehensive texture feature models were developed for improved radiomics-driven detection of prostate cancer using MP-MRI. Using a comprehensive set of texture features and a feature selection method, optimal texture feature models were constructed that improved the prostate cancer auto-detection significantly compared to conventional MP-MRI texture feature models.

## Background

Prostate cancer is the most common form of cancer diagnosed in North American men, with roughly 23,500 new cases in 2014 in Canada [1] and 233,000 new cases in 2014 in the United States [2]. Furthermore, prostate cancer is the third leading cause of cancer death in Canadian men with an estimated 4,000 deaths [1], and second leading cause of cancer death in men in the United States with an estimated 29,480 deaths in 2014 [2]. Given that the median patient survival time for metastatic prostate cancer ranges from 12.2 to 21.7 months [3], early diagnosis of clinically significant prostate cancer would have significant benefits to patient care. This is particularly true given that the five-year survival rate after diagnosis for patients with prostate cancer at the non-metastatic stage is 96 % in Canada [4].

In the current clinical model, men with positive digital rectal exam (DRE) and elevated prostate-specific antigen (PSA) require multicore random biopsies for risk stratification. However, there is an ongoing controversy about the role of prostate PSA as a screening test in prostate cancer. Two recent major randomized clinical trials [5, 6] have demonstrated that PSA screening contains a significant risk of overdiagnosis for prostate cancer where it is estimated that 50 % of screened men are diagnosed with prostate cancer. This leads to painful needle biopsies and subsequent potential overtreatment [5–8]. Moreover, it has become increasingly clear that carrying out prostate biopsy procedures escalates hospital admission rates due to infectious complications, regularly resulting in discomfort and possible sexual dysfunction while with the chance of the needle missing cancerous tissue [9–11]. Nevertheless, PSA testing has proven to reduce prostate cancer mortality by 20–30 % at long-term follow-ups [10]. Therefore, the PSA testing remains an important biomarker in diagnosing prostate cancers that are clinically significant. The remaining challenge is how to improve the prostate cancer diagnosis to reduce the overdiagnosis of clinically insignificant cancers.

Automatic detection of prostate cancer as part of a clinical decision support system can potentially help radiologists in interpreting images more accurately. Specifically, multi-parametric MR imaging (MP-MRI), which combines two or more of T2-weighted MRI (T2w), diffusion-weighted imaging (DWI), dynamic contrast enhanced imaging (DCE), and spectroscopy has been investigated as a promising approach for prostate cancer diagnosis and construction of detection algorithms [12–16]. By taking advantage of the unique quantitative information provided by each individual imaging technique, MP-MRI can exploit the different characteristics of prostate tissue to improve differentiation between cancerous and surrounding tissue. For example, cancerous tissue in the prostate gland may exhibit a moderate drop in signal in T2w [17] (which characterizes differences in transverse (spin-spin) relaxation time of tissue), restricted diffusion in DWI [17] (which characterizes diffusion of water in tissue), earlier onset time, higher peak, and shorter peak time in DCE [18] (which characterizes the concentration of an injected gadolinium contrast agent over time as it passes into the extracellular extravascular space of the tissue). Moreover, studies have demonstrated the ability of MP-MRI to direct biopsy with MRI/Ultrasound fusion techniques [19] and to predict Gleason score [16] and tumour volume [20]. The pulse sequence that has shown the most promise is DWI in the peripheral zone and the combination of T2w and DWI in the transition zone [21, 22]. The apparent diffusion coefficient (ADC) map in particular has shown the most promise as a biomarker [16, 23–27]. Although DCE is considered as part of MP-MRI, T2w+DWI is the most common MP-MRI because it has the most diagnostic value and does not require invasive contrast agent as DCE does.

Radiologists’ interpretations of MP-MRI have shown to achieve good prostate cancer detection rates, reaching accuracies of 80 % in the peripheral zone of the prostate gland [28]. Similarly, several algorithms have been proposed for auto-detection of prostate cancer using MP-MRI setting [13–15, 29–31]. These algorithms usually compute a set of low-level features from the MP-MRI data to construct feature vectors. Next, a supervised classifier is trained using the computed feature vectors from the training cases and their associated ‘ground-truth’ labels (e.g., labeled healthy or cancerous). Finally, the trained classifier is used to classify new cases. The reported values for accuracy of cancerous versus healthy tissue classification ranges from 64 % to 89 %, depending on the feature sets and training/test data.

Different texture features have been used in the literature for automatic detection and classification of prostate cancer. Most of the reviewed methods utilized texture features that are based on one or more of the following methods: First-Order statistical method, second-order statistical methods or Co-Occurrence Matrices [32, 33], steerable Gabor filter [34], Gradient based features (e.g., Kirsch [35]), fractal based features [36], run length matrices [37], and discrete cosine transform (DCT) [38]. Different classifiers are used for classification of pixels in prostate as cancerous and healthy among which support vector machine (SVM), neural networks, naive Bayesian, and random forests are most frequently used.

The usefulness of analysis of these texture features in prostate cancer detection has been demonstrated in a variety of applications. Madabhushi et al. [29] presented the utility of combining multiple features in detecting high likelihoods of prostatic adenocarcinoma from high-resolution ex-vivo MRI (i.e., following radical prostatectomy). In this method, the following feature sets were extracted from 3D voxels to train an ensemble of classifiers: First- and second order statistical method, steerable Gabor filters, Gradient based features, and discrete cosine transform (DCT). The algorithm was applied to 5 MR prostates and while specificity was high (e.g., 98 %), the sensitivity was reported to be low (36 % - 42 %).

Duda et al. [39] defined multi-image texture analysis (MITA) to characterize prostatic tissues in MR images. In this method, multiple MRI acquisitions of corresponding slices are performed to form a database of image n-tuples. This database includes T1-weighted (DCE), T2w, and DWI of 19 patients. The process of validating any MITA consisted of contouring Regions of Interest (ROI) by a clinician. Once all the ROIs were contoured, the corresponding slices from each sequence were combined to form n-tuple images, from which texture features were extracted and concatenated in a feature vector used to train the classifiers. In addition to features used in [29], fractal-based and run length features were also used. It was shown that the MP-MRI performed better compared to pairs of MRI modalities. Although accuracies of up to 99 % was reported, the evaluation was only performed on one slice (middle slice) for each modality. Moreover, the ROIs used for classification and measuring the accuracy was considerably large (400 to 2,400 pixels).

In another study, Litjens et al. [30] introduced the use of a cascaded classifier in order to characterize benign confounders such as atrophy, inflammation, benign prostatic hyperplasia (BPH), and prostatic intra-epithilial neoplasia (PIN) as the sources of challenge to diagnose malignant prostate cancer. In this paper, the authors presented the biology behind the benign confounders and bridged it with MRI sequences. The pathology annotations were propagated to MR images by registering the whole-mount slides with MR images. Different features were extracted from different MR images; second-order statistical and Gabor features from T2w, multi-scale blobness filter from ADC images, and curve fitting and pharmacokinetic features from DCE images [31]. The maximum relevance, minimum redundancy (mRMR) feature selection technique [40] was used to determine best features for separating of cancer from three non-cancer classes (BPH, inflammation, and atrophy). A cascaded classifier was used to gradually determine whether the sample is cancerous. MRI data of 31 patients with 44 corresponding histological H&E stained slides were used to evaluate the detection algorithm; a maximum accuracy of 76.4 % was achieved.

Tiwari et al. [13] proposed a method that combines structural and metabolic imaging data for separating benign versus cancerous and high Gleason score versus low Gleason grade regions in MP-MRI that includes T2w and magnetic resonance spectroscopy (MRS). Similar set of features used in [29] was used with a random forest classifier for detection where the evaluation was performed on 29 patient studies; accuracy of 86 % was achieved.

Ozer et al. [15] extracted second-order statistical and DCT features from 19 patients’ MP-MRI (T2w, DWI, and DCE) and used two classifiers (SVM and Relevance Vector Machine (RVM) [41]) to autodetect prostate cancer. The best achieved sensitivity and specificity were 78 % and 79 %, respectively. Glaister et al. [14] studied computed high b-value DWI for localization of prostate cancer and it was found that using ultra-high b-value (≥2000*s*/*mm*^2^) improves the separability of cancerous and healthy tissues significantly.

The underlying challenge in all of these auto-detection algorithms is whether there is enough separability between the cancerous and healthy tissues in a given image. This means if the separability is poor, even sophisticated feature extraction algorithms may not have a significant effect on the accuracy of cancer detection. On the other hand, improving the separability of cancerous and healthy tissues in the images would have a significant impact on the performance of cancer auto-detection algorithms, potentially reducing the dependency on the feature extraction methods.

In this paper, we propose new MP-MRI texture feature models that in addition to T2w and conventional DWI images, incorporate computed high-b diffusion-weighted imaging (CHB-DWI) [14] and the recently proposed correlated diffusion imaging (CDI) [42]. Compared to DWI images, CHB-DWI and CDI have both shown initial promise to improve visual separability of cancerous and healthy tissues in prostate, which can lead to improved performance of the proposed MP-MRI texture feature models for detecting prostate cancer. One aspect of the proposed MP-MRI texture feature models is to use non-invasive modalities aiming for higher usability in the clinical practice. Hence, we did not use DCE images in our models. Moreover, while most cancer detection algorithms use combined b-value images in the form of apparent diffusion coefficient (ADC) map, our proposed texture feature models utilize the individual b-value images of DWI to extract additional sets of features leading to improved accuracies. For each modality, the best feature subsets are selected based on different performance evaluation criteria (sensitivity or specificity). These best feature subsets are then combined to construct the comprehensive feature set from which the final best feature subset is selected to be used by the classifier. To the authors’ best knowledge, the proposed comprehensive texture feature models are the first that utilize all of the above-mentioned MP-MRI modalities and combine them using best feature subsets to construct an optimal texture feature model.

The proposed MP-MRI texture feature models are the first attempt in designing comprehensive quantitative feature sequences or *radiomics* as a high dimensional mineable feature space that can be used as both detection and prognostic tools for prostate cancer [43]. The proposed radiomics-driven models in this paper have been used for prostate cancer detection and they can be augmented for prognostic of prostate cancer as well. Studies on lung and head-and-neck cancer patients have confirmed the prognostic power of radiomics features when it comes to patient outcome prediction for personalized medicine [44, 45]. However, the prognostic capability of radiomics features has not been fully investigated for prostate cancer and this is a novel approach for identifying prostate tumours phenotypes.

In a previous work [46], the preliminary results for the proposed approach was reported. This paper is significantly different than the initial work as follows. First, the previous work only used T2w, ADC, CHB-DWI and CDI whereas the current approach also utilizes four b-value images. Second, in [46], only 19 features were used compared to 96 features used in this work. As it will be seen in the “Results” section, using more data (more images using b-value images and more features) makes the texture feature model more accurate, in terms of the cancer detection accuracy. Third, in the previous work, feature selection was not used while here we use feature selection for each modality and also for combination of different modalities. Feature selection allows to build a more optimal texture feature model leading to more accurate results. Finally, only five patients datasets were used in the previous work whereas 20 patients datasets have been used in this paper (6,535 cancerous and healthy tissue samples versus 40,975 samples) allowing for a better validation of the proposed texture feature models.

## Methods and materials

We propose MP-MRI texture feature models for prostate cancer detection which take advantage of abundance of data from different MR modalities to compute features used by the classifier. The goal is to combine features from each imaging modality that best separates cancerous pixels from healthy ones. In the following, we present the imaging methods used in the proposed model, the feature sets, and the proposed texture feature models. In addition, details about the image acquisition protocols and the performance measures are presented.

### Imaging methods

The main criteria for choosing imaging modalities used in the proposed texture feature models are twofold. First, images that are part of well-known radiology reporting system. Second, they are acquired non-invasively, with no need for contrast agents, and can be collected in a single imaging session. Recently, a structured 5-scale reporting system, PI-RADS, was proposed for consistent prostate MP-MRI reading [47] with subsequent studies confirming its effectiveness with respect to biopsy results [48]. PI-RADS consisst of T2w, DWI (ADC) as well as DCE images. Instead of using DCE which requires contrast agent, the proposed texture feature models use additional information available by DWI images which includes computed high b-value image, individual b-value images, and correlated diffusion images. This subsection summarizes the imaging methods used in the proposed MP-MRI feature models.

#### T2-weighted imaging (T2w)

T2w is a MR imaging modality in which the sensitivity of tissue is characterized by measuring the relaxation time (spin-spin) of the applied magnetic field. The T2w image of prostate usually shows a small reduction in signal in the cancerous tissue [17].

#### Diffusion-weighted imaging (DWI)

DWI is a promising imaging modality in which the sensitivity of tissue to Brownian motion of water molecules. The signal intensity is measured by applying pairs of opposing magnetic field gradient pulses, also known as lobe gradients [49]. The radio-frequency is excited by applying a 180 degree pulse on the phase of all the spins. The first gradient lobe, in turn, introduces a signal diphase in all the spins proportional to the gradient lobe area. The spins, then, evolve freely, divided into static spins and spins that move with respect to their relative position. The same intensity and polarity of the first gradient lobe is used again for a second gradient lobe, where all the static spins align to the 90 degree pulse and the moving spins never recovering the phase. The moving spins create higher diphase among the spins, acquiring less signal than that of the static spins. The diffusion-weighted signal, *S* is formulated as:

(1) $$ S = S_{0}e^{-bD}  $$

where *S*_0_ is the signal intensity without the diffusion weighting. The signal loss due to spins diphase, according to Stejskal-Tanner sequence, can be controlled by *b*, which consists of amplitude and duration of the diffusion pulses, gradient intensity and the time between the two pulses as well as the gyromagnetic ratio, and *D* represents the strength of the diffusion. The diffusion-weighted image (S) is usually generated with different *b* values which can be used to estimate apparent diffusion coefficient map (ADC) using the least-squares or maximum likelihood strategies [49]. The cancerous tissue in ADC is usually represented by a darker intensity compared to the surrounding tissue.

#### Computed high-b diffusion-weighted imaging (CHB-DWI)

Previous research has shown that high b-value DWI images (e.g., b-values greater than 1,000 *s*/*mm*^2^) allow for increased delineation between tumours and healthy tissues [14, 50] which makes the prostate cancer detection more robust. Nevertheless, due to hardware limitations, most MRI machines in practice do not produce DWI with b-values higher than 1,500 *s*/*mm*^2^ for prostate imaging. CHB-DWI is an alternative approach to obtain high-b DWI in which a computational model is used to reconstruct DWI at high b-values using low b-value DWI acquisitions [14, 51]. For our experiments, we constructed CHB-DWI with b-value at 2000*s*/*mm*^2^ using a Bayesian model with the same least squares estimation technique used for ADC, extrapolating to the b-value of 2000*s*/*mm*^2^.

#### Correlated diffusion imaging (CDI)

CDI [42] is a new diffusion magnetic resonance imaging modality, which takes advantage of the joint correlation in signal attenuation across multiple gradient pulse strengths and timings to not only reduce the dependency on the way diffusion gradient pulses are applied, but also improve delineation between cancerous and healthy tissue. The effectiveness of the delineation process depends on the models of the different types of tissue, since tumorous tissue has been empirically demonstrated to generate higher greyscale intensities at higher b-values. As such, in constructing CDI, these properties are exploited where the utilized b-values are adjusted for a given application. The local correlation of signal attenuation across all b-values within a local sub-volume is calculated to better represent the overall characterization of the water diffusion properties of the tissue. The CDI signal is obtained via signal mixing as follows [42]:

(2) $$\begin{array}{@{}rcl@{}}  CDI(x) &=& \int\ldots\int^{b_{n}}_{b_{0}} S_{0}(x){\ldots}S_{n}(x)P(S_{0}(x),\ldots,S_{n}(x)|\\ && V(x))\times {dS}_{0}(x){\ldots}{dS}_{n}(x) \end{array} $$

where *x* denotes spatial location, *b*_*i*_ represents b values, *S* denotes the acquired signal, *P* denotes the conditional joint probability density function, and *V*(*x*) denotes the local subvolume around *x*.

### Feature extraction

In order to separate the cancerous tissue from the healthy one, a set of features is calculated on a given MR imaging modality (i.e., T2w, DWI, CHB-DWI, CDI, and individual b-value images). We incorporate four well-known classes of texture features used in different studies to separate cancerous and healthy tissues in prostate. These features include first- and second-order statistical features (Haralick [32, 33]), steerable Gabor filter features [34], and Kirsch filter features [35]. The first-order statistical features include mean and standard deviation of grey-level intensity, skewness, and kurtosis. Second-order statistical features such as entropy and contrast are extracted from the gray-level co-occurrence matrix (GLCM) in four directions: 0 °, 45 °, 90 °, and 135 °. These texture features include 18 features in each direction generating a total of 72 features. Gabor features includes 12 features from three scales and four orientations and Kirsch features include the maximum gradient in eight directions. As a result, the proposed MP-MRI texture feature models consist of a total of 96 features for each imaging modality: four from first-order and 72 from second-order statistical features, eight from Kirsch, and 12 from Gabor filters. Table 1 summarizes all features used in the proposed texture feature models.

**Table 1:** Summary of textural features used in the feature model

Feature class	Feature
First-order statistical features	Mean, Standard deviation
	Skewness
	Kurtosis
Second-order statistical	Energy, Contrast
features (Haralick)	Correlation, Variance
	Inverse difference moment
	Sum average, Sum variance
	Sum entropy, Entropy
	Difference variance
	Difference entropy
	Information measure of correlation
	Homogeneity, Autocorrelation
	Dissimilarity, Cluster shade
	Cluster prominence
	Maximum probability
Gabor filters	3 scales and 4 orientations
Kirsch filters	8 directions

### Texture feature model

**Fig. 1:**
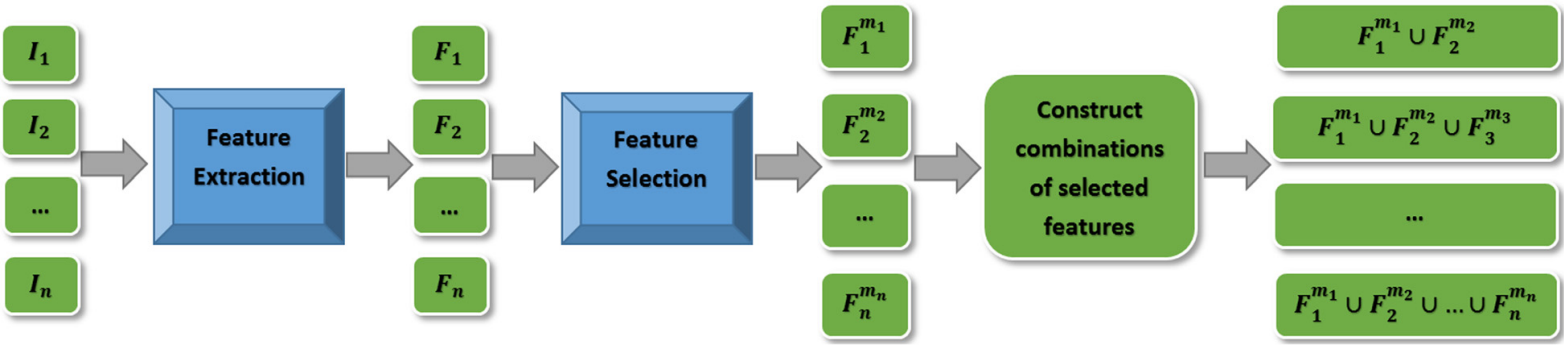
Block diagram of the proposed texture feature models
- *I*_2_ = ADC
- *I*_3_ = CHB-DWI: b-value at 2000*s*/*mm*^2^
- *I*_4_ = CDI
- *I*_5_=*b*_1_: b-value at 0*s*/*mm*^2^
- *I*_6_=*b*_2_: b-value at 100*s*/*mm*^2^
- *I*_7_=*b*_3_: b-value at 400*s*/*mm*^2^
- *I*_8_=*b*_4_: b-value at 1000*s*/*mm*^2^

Figure 1 shows the block diagram of the proposed texture feature models. The goal is to incorporate information from different sets of images to construct radiomics features; a high-dimensional feature space that can be mined for different purposes such as detection or prognosis of cancer. Similar to conventional MP-MRI, the proposed feature models include T2w and ADC modalities. They also incorporate CHB-DWI, which has been shown to increase separation between healthy and cancerous tissue. As discussed in Section “Correlated diffusion imaging (CDI)”, as a new diffusion magnetic resonance modality, CDI has shown promise in separating healthy tissue from cancerous one. Although ADC incorporates all b-value images implicitly, individual b-value images may contain information to help further distinguish healthy tissues from cancerous tissues. Therefore, we also incorporate four b-value images into our proposed texture feature models. The following lists all the imaging modalities used by the proposed texture feature models for prostate cancer detection:

- *I*_1_ = T2w

For each modality, *I*_*i*_, from the list above, the features described in Table 1 are calculated for a local window (e.g., 3×3 pixels) sliding on the prostate gland. Each window is labeled either a tumour or non-tumour voxel. For each imaging modality *I*_*i*_, this gives a feature vector *F*_*i*_.

For each voxel in each image, the feature extraction function produces 96 features. A feature selection algorithm determines a subset of features that contribute the most to the separability of classes (e.g., cancerous vs. non-cancerous tissues). This allows us to use the best features for each imaging modality when building the final texture feature models. The feature selection algorithms usually require the number of best features to be selected. For a given imaging modality *I*_*i*_, to determine the optimal number of features *m*_*i*_, we perform an exhaustive search over the feature space to evaluate the performance of any number of features. This allows us to select *m*_*i*_ features as the feature vector $F^{m_{i}}_{i}$ that produces the best results for a given imaging modality *I*_*i*_.

To evaluate the performance of a given number of features, the accuracy or area under curve (AUC) for receiver operating characteristic (ROC) curve of the classification is usually used. Cancer cells in prostate usually constitute a small fraction of the entire prostate gland (i.e., around 1 %). This means that an accuracy of an algorithm may be very high (e.g., 0.90) while it is unable to correctly locate the cancerous cells (i.e., low sensitivity). On the other hand, depending on the clinical procedures, different levels of sensitivity or specificity may be required. For example, for cancer screening programs, high sensitivity (e.g., 0.90) is required where a moderate specificity (e.g., 0.60) is deemed to be adequate. On the other hand, for a procedure such as radical prostactomy, a high specificity (e.g., 0.99) with moderate sensitivity (e.g., 0.60) is necessary to avoid unnecessary surgery. As a result, when choosing the best feature subset, it is important to consider different clinical scenarios by considering different performance evaluation criteria for feature selection.

To determine the best feature subsets, we examine two scenarios where in each scenario, it is assumed that either sensitivity or specificity has a higher priority in the performance evaluation of the proposed texture feature models. As it will be seen in the results section, depending on performance criteria used, the texture feature models produce different results.

Once the best feature subsets for each imaging modality was determined, the next step is to combine them to build different texture feature models (TFM) as follows:

- TFM _1_ = T2w+ADC
- TFM _2_ = T2w+ADC+CHB-HBV
- TFM _3_ = T2+CDI
- TFM _4_ = T2w+ADC+CDI
- TFM _5_ = T2w+ADC+HBV+CDI
- TFM _6_ = T2w+ADC+HBV+CDI+ *b*_1_ + *b*_2_ + *b*_3_ + *b*_4_

The feature selection method is applied to each texture feature model to build the final models. At this stage, the two performance criteria (sensitivity and specificity) are used to select the final best feature subsets for each texture feature model. Algorithm 1 summarizes the texture feature model construction steps.



For feature extraction function, we used the maximum relevance, minimum redundancy (mRMR) technique [40], which is based on maximum relevance and minimum redundancy of features. In this method, the feature subset $F^{m_{i}}_{i}$ is selected to satisfy the following criteria:

(3) $$ \max D(F^{m},c), D = \frac{1}{|F^{m}|}\sum\limits_{f_{i} \in F^{m}}^{m} MI(f_{i};c)  $$

(4) $$  \min R(F^{m},c), R = \frac{1}{|F^{m}|^{2}}\sum\limits_{f_{i},f_{j} \in F^{m}}^{m} MI(f_{i};f_{j})  $$

where *F*^*m*^ is the best feature subset that we would like to find, *c* is the target class, *f* is a feature and *MI* is the mutual information function. *D* and *R* are the relevance and redundancy of features, respectively. Maximum relevance guarantees that the selected features have the highest shared information with the target class and minimum redundancy ensures that the redundant features are eliminated. For the classifier, we used the SVM implemented in [52].

The proposed radiomics-driven cancer detection models combine a plethora of data from different imaging modalities of MP-MRI to construct comprehensive texture feature models which can be used for both detection and prognosis purposes in prostate cancer.

### Image data

MRI data of 20 patients (17 with cancer and three without cancer) were acquired using a Philips Achieva 3.0T machine at Sunnybrook Health Sciences Centre, Toronto, Ontario, Canada. All data was obtained retrospectively under the local institutional research ethics board (Research Ethics Board of Sunnybrook Health Sciences Centre). For each patient, the following MP-MRI modalities were obtained (Table 2): T2w, DWI, and CDI. The patients’ age ranged from 53 to 83. Table 2 summarizes the information about the 20 patients’ datasets used in this research, which includes displayed field of view (DFOV), resolution, echo time (TE), and repetition time (TR). Images were processed in the ProCanVAS (Prostate Cancer Visual Analysis System) platform developed at Sunnybrook Research Institute, Toronto, ON, Canada. Each modality (e.g., CDI) provided 40,975 samples used for the leave-one-patient-out cross-validation of the algorithms.

**Table 2:** Description of the prostate T2w, DWI, and CDI images

Modality	DFOV (*c* *m* ^2^)	Resolution (*m* *m* ^3^)	TE (ms)	TR (ms)	
T2w	22×22	0.49×0.49×3	110	4,687	
DWI	20×20	1.56×1.56×3	61	6,178	
CDI	20×20	1.56×1.56×3	61	6,178	

### Evaluation metrics

To evaluate the performance of cancer detection algorithms, two approaches may be used: pixel-based and ROI-based. In pixel-based approach [53], small neighborhoods of pixels (e.g., 3×3) are considered to distinguish cancerous tissues from healthy ones. In other words, accuracy determines the percentages of these neighborhood that were correctly labeled as cancerous or healthy. ROI-based approach [29, 39, 54] is similar to pixel-based with the difference that it uses larger neighborhoods of pixels (e.g., 50×50) for calculating accuracy measures.

In evaluating the performance of the proposed texture feature models in this paper, we use the pixel-based approach so that the accuracy measurements are calculated more precisely. As ground-truth, all MP-MR images were reviewed and marked as healthy and cancerous tissue by a radiologist with 18 and 13 years of experience interpreting body and prostate MRI, respectively. In addition, for cases with cancer, the MP-MRI images and expert annotations were compared to the corresponding histopathology data, obtained through radical prostatectomy with Gleason score seven and above, as ground-truth to confirm the accuracy of the MP-MRI markings.

## Results

**Fig. 2:**
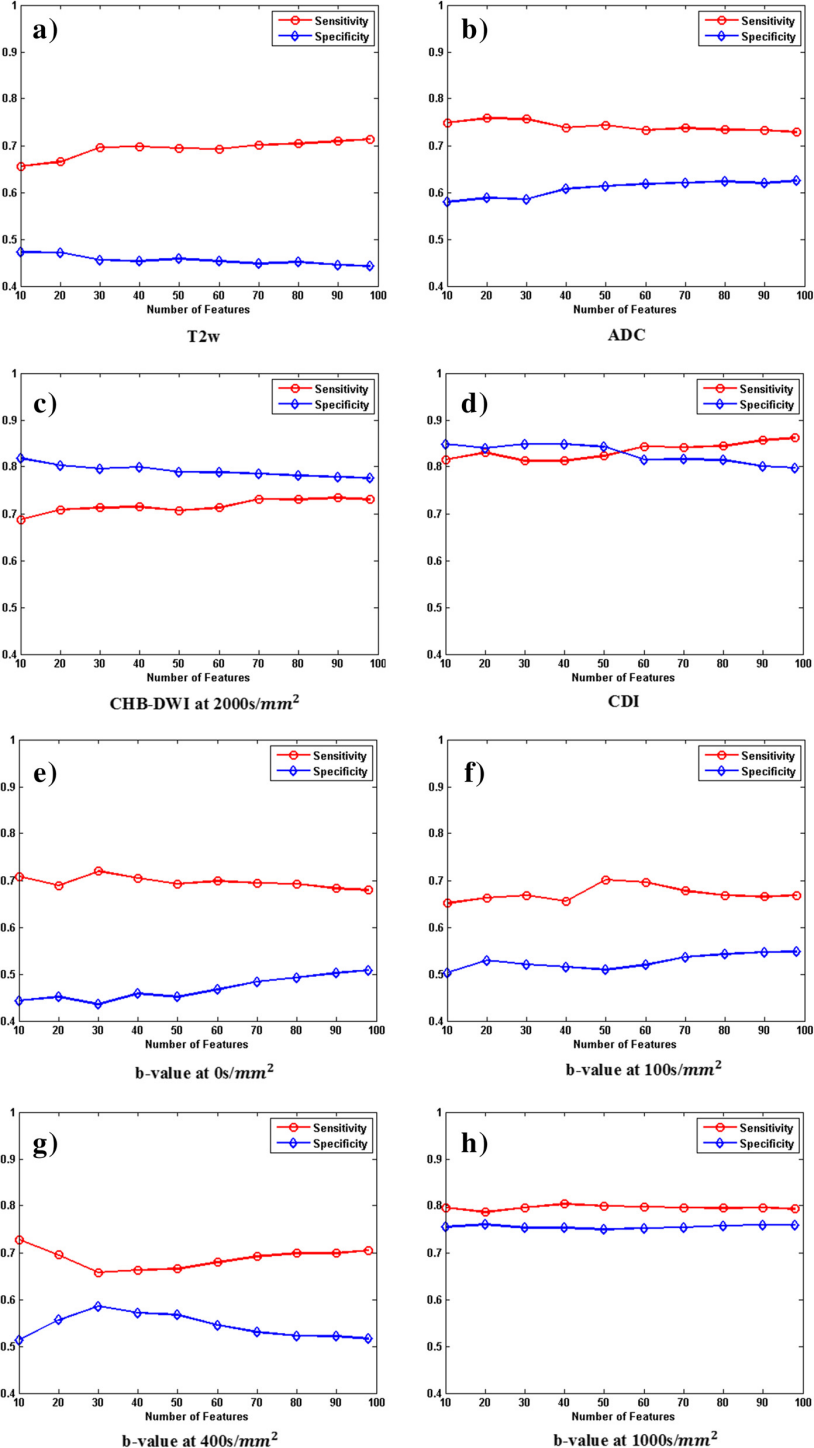
Performance results for different modalities (T2w, ADC, CHB-DWI, CDI, and 4 DWI images at different b values) across all features

Figure 2 shows sensitivity and specificity for all 8 MP-MRI modalities using different number of best features (e.g., 10 features to 96 features). For each modality, 40,975 samples (40,369 healthy and 606 cancerous samples confirmed by the radiologist) was used for the leave-one-patient-out cross-validation.

Tables 3 and 4 show the quantitative results for different modalities and combinations of modalities. Using sensitivity as feature selection criteria (Table 3), the sensitivity of the texture feature models reaches 0.86 using CDI alone. It is interesting to observe that CDI also outperforms the conventional MP-MRI (i.e., TFM _1_^1^) and combination of conventional MP-MRI and CHB-DWI (i.e., TFM _2_) (0.86 vs. 0.77 and 0.86 vs 0.69, respectively). Although CDI alone gives the best results for sensitivity (0.86), the full feature sets model (i.e., TFM _6_) produces the best results when considering specificity, accuracy, and AUC as well (0.82, 0.82, and 0.86, respectively). Comparing TFM _6_ to all other models in Table 3, at least 2 metrics out of 4 are significantly different than each of other models. For example, comparing TFM _6_ to TFME _5_, the *P* values for specificity and accuracy via Wilcoxon signed-rank test are 0.006 and 0.01, respectively.

**Table 3:** Evaluation results for prostate cancer detection: Feature selection based on Sensitivity (Results are shown with 95 *%* confidence interval)

Imaging	Number of	Sensitivity	Specificity	Accuracy	AUC
modality	features				
T2w	96	0.71 [0.54 0.89]	0.44 [0.39 0.49]	0.45 [0.40 0.50]	0.58 [0.48 0.68]
CHB-DWI	90	0.73 [0.58 0.88]	0.78 [0.71 0.85]	0.77 [0.71 0.84]	0.79 [0.73 0.85]
ADC	20	0.76 [0.64 0.88]	0.59 [0.51 0.67]	0.60 [0.52 0.67]	0.68 [0.63 0.74]
CDI	96	0.86 [0.76 0.97]	0.80 [0.75 0.85]	0.79 [0.74 0.84]	0.85 [0.81 0.90]
TFM _1_= T2w+ADC	20	0.77 [0.64 0.91]	0.57 [0.49 0.65]	0.59 [0.51 0.66]	0.68 [0.62 0.74]
TFM _2_=T2w+ADC+CHB-DWI	208	0.69 [0.54 0.84]	0.79 [0.73 0.84]	0.78 [0.73 0.84]	0.78 [0.72 0.85]
TFM _3_=T2w+CDI	196	0.85 [0.75 0.96]	0.81 [0.76 0.86]	0.80 [0.76 0.85]	0.85 [0.81 0.90]
*T* *F* *M*_4_=T2w+ADC+CDI	216	0.86 [0.76 0.96]	0.81 [0.76 0.86]	0.80 [0.76 0.85]	0.85 [0.81 0.90]
TFM _5_=T2w+ADC	300	0.86 [0.75 0.96]	0.81 [0.77 0.86]	0.81 [0.77 0.85]	0.86 [0.83 0.90]
+CHB-DWI+CDI					
TFM _6_= T2w+ADC	416	0.86 [0.75 0.97]	0.82 [0.78 0.87]	0.82 [0.78 0.86]	0.86 [0.81 0.91]
+CHB-DWI+CDI					
+ *b*_1_ + *b*_2_ + *b*_3_ + *b*_4_

**Table 4:** Evaluation results for prostate cancer detection: Feature selection based on specificity (results are shown with 95 *%* confidence interval)

Imaging	Number of	Sensitivity	Specificity	Accuracy	AUC
modality	features				
T2w	10	0.66 [0.50 0.81]	0.47 [0.42 0.53]	0.48 [0.43 0.53]	0.57 [0.48 0.66]
CHB-DWI	10	0.69 [0.52 0.86]	0.82 [0.75 0.88]	0.81 [0.75 0.87]	0.76 [0.68 0.84]
ADC	96	0.73 [0.60 0.85]	0.62 [0.55 0.70]	0.63 [0.56 0.71]	0.70. [0.64 0.76]
CDI	10	0.82 [0.69 0.94]	0.85 [0.80 0.89]	0.84 [0.80 0.88]	0.84 [0.78 0.89]
TFM _1_= T2w+ADC	110	0.72 [0.59 0.86]	0.63 [0.55 0.70]	0.64 [0.56 0.71]	0.69 [0.63 0.75]
TFM _2_=T2w+ADC	40	0.66 [0.50 0.82]	0.77 [0.71 0.83]	0.77 [0.71 0.82]	0.73 [0.65 0.81]
+CHB-DWI					
TFM _3_=T2w+CDI	20	0.78 [0.65 0.91]	0.86 [0.82 0.90]	0.86 [0.82 0.89]	0.84 [0.78 0.90]
TFM _4_=T2w+ADC+CDI	40	0.77 [0.63 0.90]	0.86 [0.82 0.90]	0.85 [0.81 0.89]	0.84 [0.79 0.89]
TFM _5_=T2w+ADC	50	0.78 [0.64 0.91]	0.86 [0.82 0.90]	0.85 [0.82 0.89]	0.84 [0.78 0.90]
+CHB-DWI+CDI					
TFM _6_=T2w+ADC	130	0.80 [0.69 0.91]	0.88 [0.85 0.92]	0.88 [0.84 0.91]	0.88 [0.83 0.93]
+CHB-DWI+CDI					
+ *b*_1_ + *b*_2_ + *b*_3_ + *b*_4_					

Table 4 shows the performance results for using specificity as performance evaluation criteria for feature selection. It is observed that compared to the previous approach (Table 3), the full feature sets model (TFM _6_) improves the specificity by 0.06 (0.88). This was expected since the performance evaluation criteria used for feature selection affects the final results. Thus, as discussed in Section “Texture feature model”, depending on the clinical scenario, one can choose different performance evaluation criteria to better suit the clinical procedure requirements. Comparing TFM _6_ to all other models in Table 4 (except for TFM _3_), at least 2 metrics out of four are significantly different than each of other models. For example, comparing TFM _6_ to TFM _5_, the *P* values for specificity and accuracy via Wilcoxon signed-rank test are 0.01. Comparing TFM _6_ to TFM _3_, the two models are significantly different with respect to AUC (*P*=0.01).

**Fig. 3:**
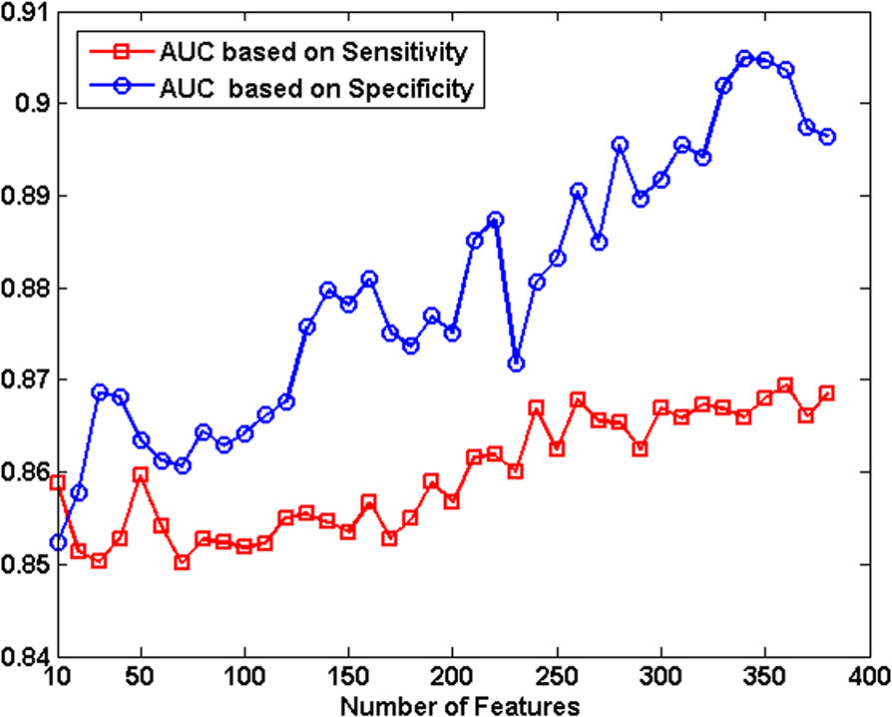
AUC based on using sensitivity and specificity as performance evaluation criteria

**Fig. 4:**
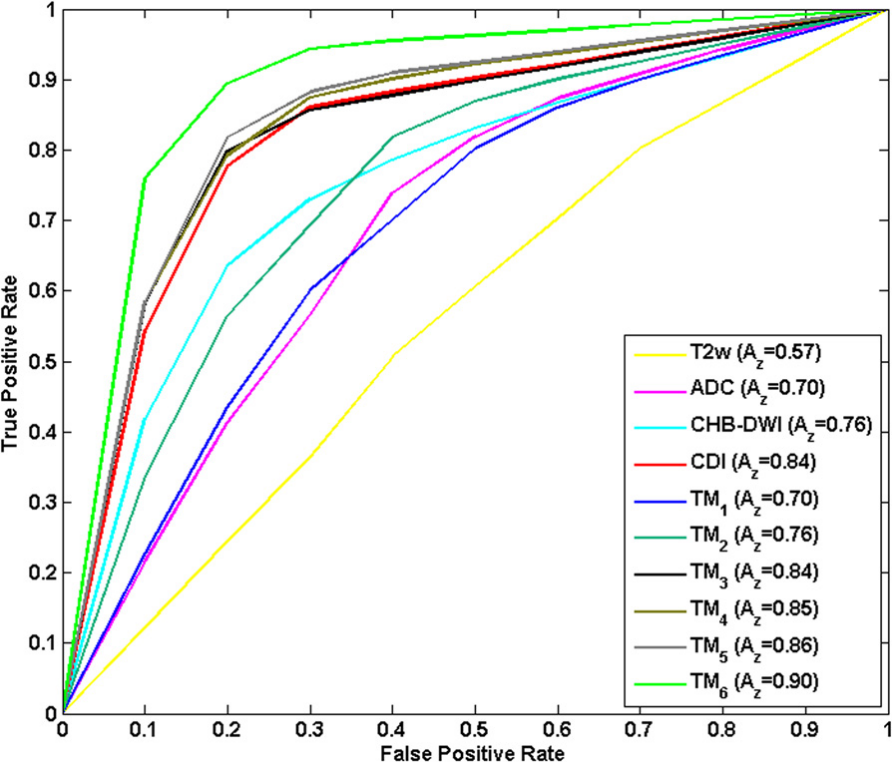
ROC for different texture feature models

Tables 3 and 4 show the result when the goal was to maximize sensitivity (Table 3) or specificity (Table 4). Figure 3 shows the combinations of all eight imaging modalities (TFM _6_) with best feature subsets based on sensitivity and specificity with the objective of maximizing for AUC. It can be seen that using specificity as performance evaluation criteria gives a higher best AUC compared to sensitivity (0.90 vs. 0.87). Figure 4 shows the ROC curves for all six models as well as individual imaging modalities discussed in Section “Texture feature model”. It is seen that the combination of all imaging modalities, TFM _6_, gives the best results in terms of AUC (0.90). This result is significantly different with respect to any other imaging modality or texture feature model where *P*<0.009.

Table 5 shows the optimal results with the target of maximizing sensitivity, specificity, or AUC. As it can be seen, choosing a target yields the best result for the selected target. Setting AUC as the target maximizes the AUC (0.90) and at the same time generates more balanced results with respect to sensitivity and specificity (0.84 and 0.86). Using sensitivity as the performance evaluation criteria maximizes the result for sensitivity (0.86). Using specificity as the performance evaluation criteria maximizes the result for specificity (0.88) and AUC (0.90), depending on the selected target.

**Table 5:** Evaluation results for prostate cancer detection: Feature selection based on Sensitivity and Specificity (Results are shown with 95 % confidence interval)

Target	Performance evaluation criteria	Sensitivity	Specificity	AUC
Sensitivity	Sensitivity	0.86 [0.75 0.97]	0.82 [0.78 0.87]	0.86 [0.81 0.91]
Specificity	Specificity	0.80 [0.69 0.91]	0.88 [0.85 0.92]	0.88 [0.83 0.93]
AUC	Specificity	0.84 [0.76 0.91]	0.86 [0.82 0.91]	0.90 [0.88 0.93]

**Fig. 5:**
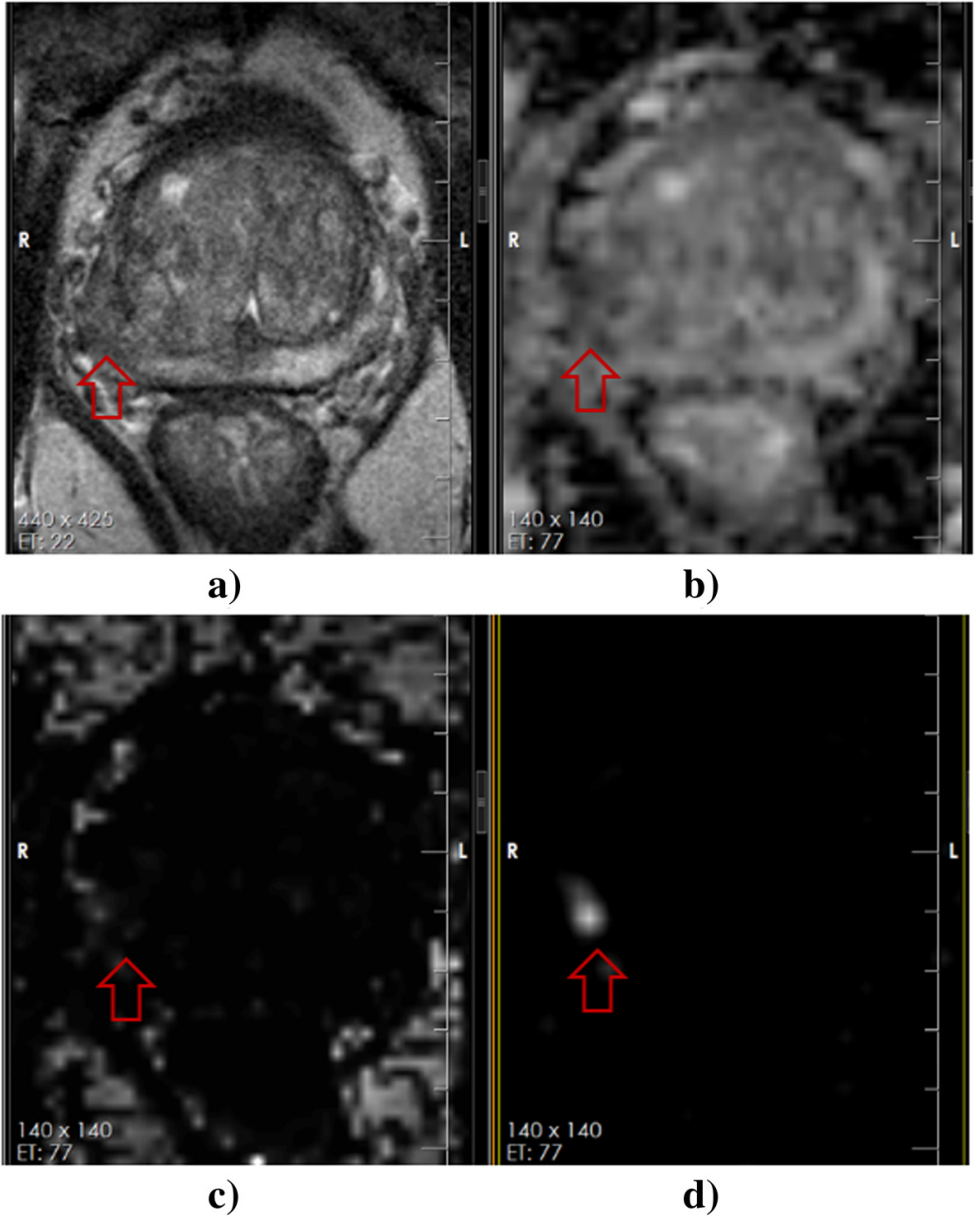
**a** T2w does not clearly show a tumour although there is mild signal alteration in the left peripheral zone (arrow). **b** ADC does not clearly show a tumour (arrow). **c** CHB-DWI of 2000 *s*/*m* *m* ^2^shows no tumour (arrow). **d** CDI clearly shows a bright nodule (arrow) corresponding to tumour

**Fig. 6:**
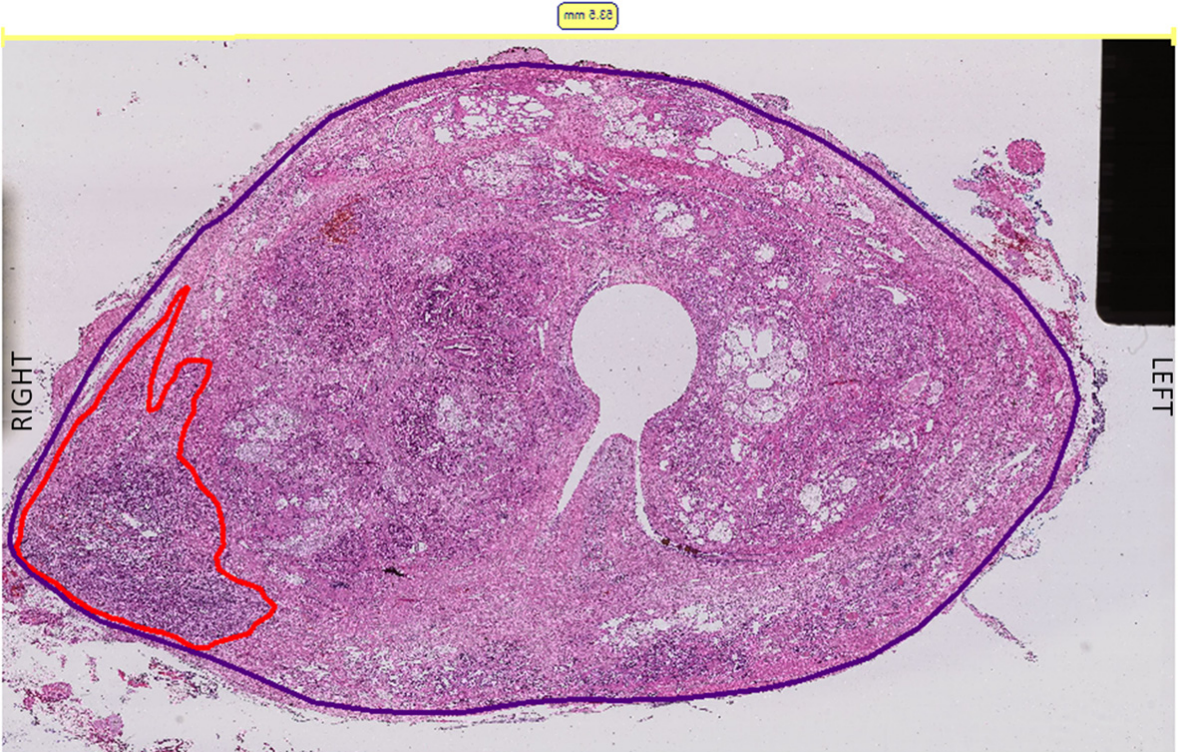
Corresponding axial hematoxylin and eosin stained tissue showing a Gleason 7 (4+3) tumor circled in red corresponding to the lesion identified best on the CDI images in Fig. 5-d

Figure 5 shows an example for all four modalities which include T2w, ADC, CHB-DWI, and CDI. As it can be seen, CDI (Fig. 5d) is the only modality that clearly shows a bright nodule where a tumour is located (confirmed by histopathology data - Fig. 6).

## Discussion

Automated prostate cancer detection has been investigated by different research groups in the field. The underlying building blocks of these algorithms consist of feature extraction and classification applied to local windows of pixels in the images. Most algorithms in the literature usually incorporate few imaging sequences into their proposed feature models. For example, the work presented in [39] used three sequences (T1w, T2w, and DCE) to build the texture feature model. In contrast, in this paper, we have taken one step further by incorporating information from all available MR imaging data which includes T2w, ADC, and different b-value images of DWI (i.e., b-values at 0, 100, 400, and 1000 *s*/*mm*^2^). Moreover, we incorporated computed high-b DWI (CHB-DWI) [14] as well as correlated diffusion imaging (CDI) [42] into our model. Adding these two extra imaging modalities enriched our texture feature models in terms of the diversity of data where 6 different models were developed and evaluated (Sections “Texture feature model” and “Results”). As a quantitative radiomics approach for prostate cancer detection, we used a comprehensive texture feature model which incorporated eight different imaging modalities where each modality contributed with its best feature subset to the ultimate texture feature model in which all modalities were combined.

One important aspect in the clinical workflow for prostate cancer detection is the targeted clinical procedure. For example, cancer screening programs impose different performance requirements compared to procedures such as radical prostatectomy. We designed the proposed texture feature model accounting for such requirements where the performance of the model can be optimized for sensitivity, specificity, or the area under the ROC curve. For example, to use the proposed texture feature model for cancer screening, sensitivity can be used as the performance evaluation criteria to steer the feature selection process which would lead to best result for sensitivity (0.86) with reasonable results for specificity (0.82). For cases where higher specificity is required, one can use specificity as the performance evaluation criteria to optimize the results for specificity (0.88) with acceptable sensitivity (0.80). Our experiments showed that using specificity as the performance evaluation criteria can also maximize the results for AUC (0.90) which leads to a balanced results for sensitivity and specificity; 0.84 and 0.86, respectively. The fact that the proposed model is flexible in terms of optimizing the results for the procedure it is used for makes it more practical. This is another novel aspect of the proposed model in this paper which to the authors’ best knowledge has not been fully explored in the literature.

The limitations of our research include a relatively limited number of datasets (20 patients) and targeting only Gleason score of seven and above. Evaluating the proposed model using a larger dataset and considering lower Gleason scores (e.g., six) will add more confidence to the reliability of the model which will be done as future work. Other limitation is that the proposed model was not assessed by clinicians to investigate whether it improves the clinical readings by radiologists. Similar to the work reported in [55], clinical assessment of the proposed auto-detection model will be performed to evaluate its effect on the clinicians’ performance. Finally, given the fact that cancerous pixels are a small fraction of the entire prostate gland, it is possible that the reported specificity results are an overestimation. A larger and more diversified dataset will help to investigate this more thoroughly.

Our proposed texture feature model incorporated CDI as one of the imaging modalities which as shown in Section “Results”, boosted the results significantly. We have developed an enhanced version of CDI, called dual-stage correlated diffusion imaging (D-CDI) which has shown promise in enhancing separability of cancerous and healthy tissue in prostate MRI compared to CDI [56]. As future work, we will incorporate D-CDI to the proposed texture feature model to investigate the effect on performance. We will also investigate developing a hybrid morphological-textural feature model for prostate cancer where in addition to texture analysis, the morphological characteristics (e.g., shape) of candidate regions are taken into account to detect cancer. A preliminary work on morphological feature model has been presented in [57] upon which we will extend and build the hybrid model. The b-value images of DWI are usually distorted due to patient movement during the image acquisition which may reduce cancer separability. We have presented preliminary results for co-registering the b-value images to compensate for patient movement [58]. As future work, we will incorporate this co-registration algorithm into our proposed texture feature models to investigate the effect on the accuracy of cancer detection. Finally, normalized entropy has been shown to be a strong predictor of patient survival rate for lung and renal cell cancers in CT images [59]. The future work will also involve investigating the efficacy of the proposed texture feature model in this paper for normalized MP-MRI entropy characterization of prostate cancer.

## Conclusion

In this paper, we introduced new multi-parametric MRI texture feature models for prostate cancer detection. Our new MP-MRI texture feature models add two new imaging modalities, computed high-b DWI and correlated diffusion imaging, to the most commonly used MP-MRI, T2w+ADC. As a quantitative radiomics approach for automatic detection of prostate cancer, a comprehensive set of texture features were calculated for the conventional MP-MRI and new MP-MRI texture feature models. A feature selection method was used to select the optimal features for each modality. Two different performance evaluation criteria were used for feature selection to reflect different clinical workflows in prostate cancer diagnosis. The best feature subsets were then combined to construct optimal texture feature models. A SVM classifier was trained via leave-one-patient-out setting to classify the new cases. The proposed MP-MRI texture feature models showed promise in accurate detection of prostate cancer.

## Endnote

^1^ The conventional MP-MRI refers to the combination of T2w and DWI, which is represented as ADC. Thus, throughout the paper, T2w+DWI and T2w+ADC are used interchangeably.
